# A Low-Cost INS-Integratable GNSS Ultra-Short Baseline Attitude Determination System

**DOI:** 10.3390/s18072114

**Published:** 2018-07-01

**Authors:** Wenyi Li, Peirong Fan, Xiaowei Cui, Sihao Zhao, Tianyi Ma, Mingquan Lu

**Affiliations:** Department of Electronic Engineering, Tsinghua University, Weiqing Building, Tsinghua, Haidian District, Beijing 100084, China; lia_lwy@live.com (W.L.); fpr09@foxmail.com (P.F.); zsh_thu@tsinghua.edu.cn (S.Z.); matianyi@mail.tsinghua.edu.cn (T.M.); lumq@tsinghua.edu.cn (M.L.)

**Keywords:** GNSS, attitude determination, ambiguity resolution, low-cost receivers, GNSS/INS integrated system

## Abstract

Traditional attitude determination using global navigation satellite system (GNSS) carrier phases is mostly applied on geodetic-grade receivers with sufficient baseline length. However, for some special applications such as mobile communication base station smart antenna attitude determination, only low-cost receivers with ultra-short baselines can be employed, and the environments are more challenging. When solving the ambiguity resolution (AR) problem with low-cost receivers, it is hard for the traditional methods in ambiguity domain to estimate float ambiguities accurately due to the large code pseudorange noises; thus, such systems fail to determine the correct ambiguities. Aiming at improving the AR success rate for ultra-short baselines attitude determination with low-cost receivers, we provide an objective function named Mean Square Residual (MSR) based on the geometrical relationship among the position spherical search space, the fractional carrier phases, and the possible ambiguities. The method can be calculated without code pseudoranges, and thus, can provide a higher AR success rate when using low-cost receivers. The corresponding analysis and acceptance test method are discussed in this contribution, and further, as an extension for more complicated urban dynamic applications, a GNSS/Inertial Navigation System (INS) integrated system is introduced. Several experiments have been conducted to verify performance.

## 1. Introduction

Attitude determination with differential global navigation satellite system (GNSS) carrier phases is performed in a wide range of applications, including surveying, vehicle active safety, and driver assistance system and aircraft/ship attitude determination.

Traditional GNSS-based attitude determination applications are mostly implemented with geodetic-grade receivers and antennas with sufficient length of baselines and are generally deployed in open areas that are less affected by multipaths. However, for some special scenarios, due to limits of budget and load capacity, only small and light-weight, low-cost receivers and antennas are applicable. For example, for mobile communication base station smart antenna attitude determination, because of the large application demand, manufacturers prefer the use of low-cost devices, and usually, the GNSS antennas are installed right next to the base station within a small area, bringing problems like signal blockage and strong multipaths. Besides that, some other applications like attitude determination for urban multi-rotor unmanned air vehicle (UAV) have extra requirement of instantaneity. As a common ground for the above-mentioned applications, because of the limited installation space, the antennas are usually placed at a short distance, forming an ultra-short baseline.

Carrier phase measurements have high precision but are ambiguous because of the unknown integer cycles between the satellite and receiver, namely, ambiguities. Integer ambiguity resolution (AR) is the key to the success of attitude determination with carrier phases. Traditional AR algorithms are generally carried out in the ambiguity domain, which comprises two major steps [1,2]: Firstly, the baseline vector and the float values of ambiguities are estimated by solving the equations of code pseudorange and carrier phase measurements. Then, a search is done for the correct integer ambiguities in a search space centered around the estimated float values. The most popular searching method is the Least-squares AMBiguity Decorrelation Adjustment (LAMBDA) method [3]; for attitude determination applications, the success rate can be further improved by introducing tight or soft baseline length constraints into the least-squares objective function [4,5,6]. The performance of the LAMBDA-based methods greatly depends on the accuracy of the float ambiguity estimation which is mainly predominated by the code pseudorange precision [7,8]; thus, LAMBDA methods are more inclined to be performed with geodetic-grade receivers in open areas based on multi-epoch stable observation [9].

However, for the above-mentioned special attitude determination applications with low-cost receivers, given the challenges such as single frequency, larger code pseudorange noises and inferior performance of low-cost receivers, it is harder for the traditional LAMBDA methods to accurately estimate float ambiguities [10,11], which makes the least-squares searching objective function less viable, and thus, leads to an increasing probability of wrong-fixing. For real-time dynamic applications in complicated environments, due to frequent satellite number changes and cycle slips, the methods aiming at fixing the ambiguities based on multi-epoch observation are not applicable, and thus, the single-epoch methods are better suited to this situation. Moreover, the multipath effect is also an inevitable challenge for complicated environments. Compared with geodetic-grade receivers, low-cost receivers are more sensitive to multipath effects, which may produce pseudorange noise at a magnitude up to 10 m or even higher.

To overcome these defects, some researchers have shifted their focus to algorithms in the position domain, among which the Ambiguity Function Method (AFM) [12] is the most famous. Some others have tried to solve the AR problem without pseudoranges. Chen et al. proposed an improved LAMBDA method by substituting the traditional float ambiguities with a set of float ambiguity candidates derived by carrier phases, only to reduce the effect of the inaccurate float ambiguities [13]. However, both AFM and Chen’s method result in high computational complexity.

Aiming at improving the success rate of single-epoch attitude determination with low-cost receivers, we explore the relationship among the baseline vector search space, the carrier phase measurements and ambiguities, and propose a single-epoch algorithm in angle domain. With the knowledge of the known baseline length, a 2-D traversal in the angle domain is made to obtain a set of candidate position points. Our objective function is designed based on the fractional differences between the carrier phase measurements and the calculated geometric distances of each candidate point (measured in unit of cycle). That way, we can pick out a best candidate position point and get the ambiguities accordingly, without the use of code pseudoranges. The method is executed with fractional parts of carrier phases only, and thus, is insensitive to cycle slips. Taking the balance between the stability and computational efficiency into consideration, we also provide an efficient angle traversal method and deduce the upper bounds of angle search step lengths.

For dynamic applications in urban environments, considering the availability, output rate and accuracy of ultra-short baseline attitude determination, a better solution is to integrate GNSS with an Inertial Navigation System (INS) [11,14]. For INS, calibration is indispensable, and one simple way is to use GNSS results to make the calibration [15]. The focus of this contribution is to develop a reliable single-epoch, low-cost, GNSS-based, ultra-short baseline attitude determination algorithm with a higher success rate, which can be used alone or for INS calibration. As an extension, we propose a GNSS/INS integrated system for urban dynamic situations.

After a brief review of measurement models, the principle of the proposed algorithm in the angle domain is presented, followed by an algorithm performance analysis and a discussion of the corresponding acceptance method. Then, the process of the proposed GNSS attitude determination system is given, along with an extended system integrated with INS. Finally, the experimental results under different environments are presented to verify performance.

## 2. Measurement Models

**Figure 1:**
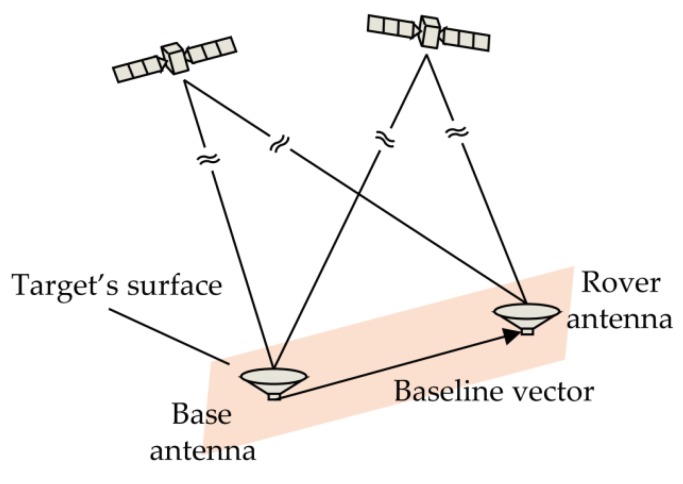
Basic principle of attitude determination with GNSS.

The basic principle of attitude determination with GNSS, as shown in Figure 1, is to measure the precise relative position between multiple antennas fixed on the target surface, and then to determine the attitude of the target. If the baseline length is known, the baseline vector $r_{rb}$ in East-North-Up (ENU) coordinate whose origin point is the base antenna can be expressed as a function of the heading φ and pitch θ:

(1) $$r_{rb}=\left[\begin{array}{c} e_{rb}\\ n_{rb}\\ u_{rb} \end{array}\right]=\left[\begin{array}{c} l\sin\left(\varphi \right)\cos\left(\theta \right)\\ l\cos\left(\varphi \right)\cos\left(\theta \right)\\ l\sin\left(\theta \right) \end{array}\right]$$

where l is the known baseline length, $e_{rb}$, $n_{rb}$ and $u_{rb}$ are the east, north and up components of $r_{rb}$ respectively.

The undifferenced code pseudorange and carrier phase measurements can be modeled as [16]:

(2) $$P_{r}^{i}=\rho_{r}^{i}+cdt_{r}-cdT^{i}+T_{r}^{i}+I_{r}^{i}+E_{r}^{i}+MP_{r,P}^{i}+\varepsilon_{r,P}^{i}$$

(3) $$\lambda \Phi_{r}^{i}=\rho_{r}^{i}+cdt_{r}-cdT^{i}+T_{r}^{i}-I_{r}^{i}+E_{r}^{i}-\lambda N_{r}^{i}+MP_{r,\Phi }^{i}+\varepsilon_{r,\Phi }^{i}$$

where $P_{r}^{i}$ and $\Phi_{r}^{i}$ are the code pseudorange and carrier phase measurements between satellite i and receiver r, respectively, λ is the wavelength, $\rho_{r}^{i}$ is the geometric range, c is the speed of light, $dt_{r}$ and $dT^{i}$ are the clock biases of the receiver and satellite, respectively, $I_{r}^{i}$ is the ionospheric delay, $T_{r}^{i}$ is the tropospheric delay, $E_{r}^{i}$ is the ephemeris error, $N_{r}^{i}$ is the unknown integer ambiguity, $MP_{r,P}^{i}$ and $MP_{r,\Phi }^{i}$ are code and carrier multipath errors, respectively, and $\varepsilon_{r,P}^{i}$ and $\varepsilon_{r,\Phi }^{i}$ represent all other errors that cannot be modeled.

To eliminate the effect of the clock bias of the satellite i, we can do single differencing between two antennas; for short-baseline cases, ephemeris and atmospheric errors can also be eliminated by single differencing, and we can get the single differenced (SD) measurements:

(4) $$\Delta P_{rb}^{i}=P_{r}^{i}-P_{b}^{i}=\Delta \rho_{rb}^{i}+c\Delta dt_{rb}+\Delta MP_{rb,P}^{i}+\Delta \varepsilon_{rb,P}^{i}$$

(5) $$\lambda \Delta \Phi_{rb}^{i}=\lambda \Phi_{r}^{i}-\lambda \Phi_{b}^{i}=\Delta \rho_{rb}^{i}+c\Delta dt_{rb}-\lambda \Delta N_{rb}^{i}+\Delta MP_{rb,\Phi }^{i}+\Delta \varepsilon_{rb,\Phi }^{i}$$

Further, to remove the effects of clock biases of receivers, double differencing between two satellites are done, forming the following double differenced (DD) measurements:

(6) $$\nabla \Delta P_{rb}^{ij}=\Delta P_{rb}^{i}-\Delta P_{rb}^{j}=\nabla \Delta \rho_{rb}^{ij}+\nabla \Delta MP_{rb,P}^{ij}+\nabla \Delta \varepsilon_{rb,P}^{ij}$$

(7) $$\lambda \nabla \Delta \Phi_{rb}^{ij}=\lambda \Delta \Phi_{rb}^{i}-\lambda \Delta \Phi_{rb}^{j}=\nabla \Delta \rho_{rb}^{ij}-\lambda \nabla \Delta N_{rb}^{ij}+\nabla \Delta MP_{rb,\Phi }^{ij}+\nabla \Delta \varepsilon_{rb,\Phi }^{ij}$$

For traditional AR methods in the ambiguity domain, the above DD code pseudorange and carrier phase measurement equations are usually combined to obtain the baseline vector ${\hat{r}}_{rb}$ and the float ambiguity estimation $\nabla \Delta \overset{⌣}{N}$. Then, for the LAMBDA method, the following objective function is used to select the integer ambiguity solution $\nabla \Delta \overset{⌣}{N}$ based on least-squares principle [2].

(8) $$q\left(\nabla \Delta N\right)={\left\|\nabla \Delta \hat{N}-\nabla \Delta N\right\|}_{Q_{\nabla \Delta \hat{N}}}^{2}$$

(9) $$\nabla \Delta \overset{⌣}{N}=\underset{\nabla \Delta N\in {\mathbb{Z}}^{n}}{\arg\min}\;q\left(\nabla \Delta N\right)$$

where $Q_{\nabla \Delta \hat{N}}$ is the covariance matrix of the estimated float ambiguities.

For the above ILS problem, the most common ambiguity acceptance test is the ratio-test [17,18], which is defined as follows

(10) $$\mathrm{Accept}\;\nabla \Delta \overset{⌣}{N}\;\mathrm{iff}:\;\frac{q\left(\nabla \Delta \overset{⌣}{N}\right)}{q\left(\nabla \Delta {\overset{⌣}{N}}^{\prime }\right)}\geq thres_{q}$$

where $\nabla \Delta {\overset{⌣}{N}}^{\prime }$ is integer ambiguity vector of the second smallest value of q(∇ΔN), and $thres_{q}$ is the tolerance threshold.

From (8), we may observe that the float ambiguity estimation $\nabla \Delta \hat{N}$ plays an important role as a referenced center in the objective function q(∇ΔN), and thus, determines the success rate of the AR problem. For low-cost receivers under challenging environments, due to the large pseudorange noises, float ambiguities $\nabla \Delta \hat{N}$ cannot be estimated accurately, thus causing difficulties for the search and latter acceptance test judgment. For attitude determination applications with known baseline length, the objective function (8) can be modified by introducing additional term of tight or soft constraint [4,19], but the float ambiguity estimation still predominates the function.

## 3. Basic Idea of Ambiguity Resolution in Angle Domain

To get rid of the effect of float ambiguity estimation, in this contribution, we focus on the single-epoch AR algorithm in position domain, particularly, in angle domain. Furthermore, since the quality of code pseudoranges is hard to determine definitively for low-cost receivers, code pseudoranges are no longer used, which means only carrier phase measurements are employed in our algorithm.

To better understand the relationship among the baseline vector search space, the carrier phase measurements, and ambiguities, a formula variant is made (below). We can reserve only the fractional parts of the carrier phase measurements and rearrange the DD carrier phase measurement (7), as follows:

(11) $$\frac{1}{\lambda }\nabla \Delta \rho_{rb}^{ij}=\nabla \Delta {\tilde{\Phi }}_{rb}^{ij}+\nabla \Delta {\bar{N}}_{rb}^{ij}-\frac{1}{\lambda }\left(\nabla \Delta MP_{rb,\Phi }^{ij}+\nabla \Delta \varepsilon_{rb,\Phi }^{ij}\right)$$

where $\nabla \Delta {\tilde{\Phi }}_{rb}^{ij}$ is the fractional part of the DD carrier phase measurement whose range is from −0.5 to 0.5 cycle, and $\nabla \Delta {\bar{N}}_{rb}^{ij}$ is the DD ambiguity corresponding to $\nabla \Delta {\tilde{\Phi }}_{rb}^{ij}$. The SD and DD geometric ranges can be approximated as:

(12) $$\frac{1}{\lambda }\Delta \rho_{rb}^{i}\approx -e^{i}{}^{T}\cdot \frac{1}{\lambda }r_{rb}$$

(13) $$\frac{1}{\lambda }\nabla \Delta \rho_{rb}^{ij}\approx {\left(e^{j}-e^{i}\right)}^{T}\cdot \frac{1}{\lambda }r_{rb}$$

where $e^{i}$ is the unit line-of-sight (LOS) vector pointing from the receiver to the satellite i. If we substitute (13) for (11), and ignore the multipath and other noises, we get:

(14) $$\frac{\nabla \Delta {\tilde{\Phi }}_{rb}^{ij}+\nabla \Delta {\bar{N}}_{rb}^{ij}}{\left\|e^{j}-e^{i}\right\|}\approx \frac{{\left(e^{j}-e^{i}\right)}^{T}}{\left\|e^{j}-e^{i}\right\|}\cdot \frac{1}{\lambda }r_{rb}$$

**Figure 2:**
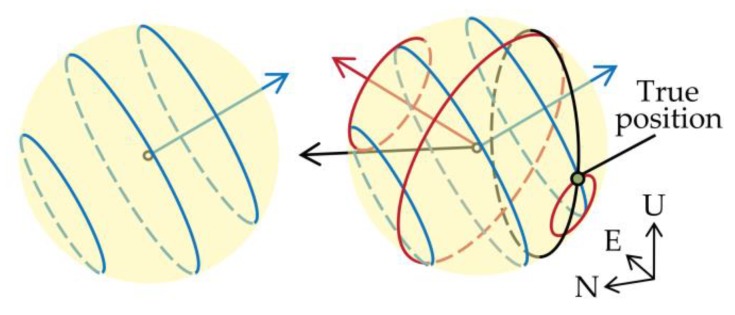
Relationship between the baseline vector and the fractional parts of the DD carrier phases.

The right side of (14) is the baseline vector $\frac{1}{\lambda }r_{rb}$’s projection on $\left(e^{j}-e^{i}\right)$ direction, and the value of the projection is given by the left side, which is composed of the fractional part of the DD carrier phase measurement and the unknown DD ambiguity. Figure 2 shows an error-free example for the relationship between the baseline vector (measured in unit of cycle) and the fractional parts of the DD carrier phase measurements in the ENU coordinate. The baseline length is 0.4 m, and the true pitch and heading are 0° and 240°, respectively. The blue, black, and red arrows represent the directions $\left(e^{1}-e^{2}\right)$,$\left(e^{1}-e^{3}\right)$ and $\left(e^{1}-e^{4}\right)$, respectively, and the elevations and azimuths of the referenced satellite and other satellites are listed in Table 1. With knowledge of the baseline length, the possible baseline vectors fall into a spherical surface with a radius of the baseline length. For each differenced LOS vector $\left(e^{j}-e^{i}\right)$, according to (14), a set of parallel circles perpendicular to the vector can be drawn, each of which is determined by the fractional part of the DD carrier phase and possible integer DD ambiguity. If no errors exist, different sets of circles will intersect, as shown in Figure 2, and the intersection point is the true value of the baseline vector. However, affected by the subsistent noises, these circles may not intersect exactly at the same point. Therefore, our solution is to discretize the spherical surface into a set of candidate points by a 2-D traversal in angle space and select an approximate intersection point. The criterion for selecting the approximate intersection point in our algorithm is to see how far the point is from the closest circle of each DD measurement.

By varying in angle space, we obtain a candidate position point set $\left\{r_{rb,k}\right\}$ on the spherical surface. For a candidate position point $r_{rb,k}$, we can calculate its distance to the nearest circles for all the pairs of satellites. However, when calculating the “distances”, considering the computational complexity, we employ the vertical distances to the circle planes instead of the direct distances to the circle edges. For each DD geometric range, we can process this in the following way:

(15) $$\begin{array}{ll} \frac{1}{\lambda }\nabla \Delta \rho_{rb,k}^{ij} & =\nabla \Delta {\tilde{\Phi }}_{rb}^{ij}+\left(\frac{1}{\lambda }\nabla \Delta \rho_{rb,k}^{ij}-\nabla \Delta {\tilde{\Phi }}_{rb}^{ij}\right)\\ & =\nabla \Delta {\tilde{\Phi }}_{rb}^{ij}+round\left(\frac{1}{\lambda }\nabla \Delta \rho_{rb,k}^{ij}-\nabla \Delta {\tilde{\Phi }}_{rb}^{ij}\right)+rem\left(\frac{1}{\lambda }\nabla \Delta \rho_{rb,k}^{ij}-\nabla \Delta {\tilde{\Phi }}_{rb}^{ij}\right) \end{array}$$

Again, we substitute (15) into $\frac{1}{\lambda }r_{rb,k}$’ s approximate expression in (13) form, we can get the baseline vector $\frac{1}{\lambda }r_{rb,k}$’s projection expression, with actual measurement noises as follows:

(16) $$\frac{{\left(e^{j}-e^{i}\right)}^{T}}{\left\|e^{j}-e^{i}\right\|}\cdot \frac{1}{\lambda }r_{rb,k}=\frac{\nabla \Delta {\tilde{\Phi }}_{rb}^{ij}+round\left(\frac{1}{\lambda }\nabla \Delta \rho_{rb,k}^{ij}-\nabla \Delta {\tilde{\Phi }}_{rb}^{ij}\right)}{\left\|e^{j}-e^{i}\right\|}+\frac{rem\left(\frac{1}{\lambda }\nabla \Delta \rho_{rb,k}^{ij}-\nabla \Delta {\tilde{\Phi }}_{rb}^{ij}\right)}{\left\|e^{j}-e^{i}\right\|}$$

The term containing the DD fractional carrier phase and the integer in (16) is the distance from the original point to the nearest circle plane, and then, the vertical distance to the nearest circle plane can be easily obtained by the remaining term:

(17) $$d_{k}^{ij}=\left|\frac{rem\left(\frac{1}{\lambda }\nabla \Delta \rho_{rb,k}^{ij}-\nabla \Delta {\tilde{\Phi }}_{rb}^{ij}\right)}{\left\|e^{j}-e^{i}\right\|}\right|$$

Since the actual carrier phase measurement $\nabla \Delta {\tilde{\Phi }}_{rb}^{ij}$ contains error, which is scaled by $1/\left\|e^{j}-e^{i}\right\|$ in the above distance calculation formula, we can multiply the calculated distance by a weight $\left\|e^{j}-e^{i}\right\|$ before making statistics for all the distances. Thus, we can define a so-called mean square residual (MSR) as the objection function. For the candidate position point $r_{rb,k}$, its MSR value is:

(18) $$MSR_{k}={\left\|v_{k}\right\|}_{2}$$

where ${\left\|\cdot \right\|}_{2}$ is the 2-norm operator (mean square function), $v_{k}={\left(v_{k}^{21},v_{k}^{31},\cdots ,v_{k}^{n1}\right)}^{T}$, where the expression of $v_{k}^{ij}$ is:

(19) $$v_{k}^{ij}=rem\left(\frac{1}{\lambda }\nabla \Delta \rho_{rb,k}^{ij}-\nabla \Delta {\tilde{\Phi }}_{rb}^{ij}\right)$$

It is reasonable to believe that the best candidate position point is the one with the smallest MSR, and once decided, the ambiguities can be decided by the nearest circles of the point:

(20) $$\nabla \Delta {\bar{N}}_{rb}^{ij}=round\left(\frac{1}{\lambda }\nabla \Delta \rho_{rb,k}^{ij}-\nabla \Delta {\tilde{\Phi }}_{rb}^{ij}\right)$$

From the calculation formula, we can find the essence of MSR is to judge the fractional differences between the calculated geometric ranges and the carrier phase measurements. For low-cost receivers, carrier phases are less effected by multipath than code pseudoranges. Compared to traditional methods in ambiguity domain using code pseudoranges, our algorithm uses carrier phases only. The algorithm is more focused on the relationship between the position search space and the satellite geometric distribution, which leads to a higher success rate for AR problem; thus, the main advantage of the proposed method is the applicability to low-cost receivers.

## 4. Candidate Position Point Set

In this section, the derivation of the candidate position point set, or specifically, how to discretize the spherical search space more efficiently, is discussed. The aim is to minimize the number of the candidate positions under the precondition of the algorithm stability, so as to lighten the calculation burden.

To discretize the spherical search space more efficiently, we firstly vary θ at a certain search step to get a set of horizontal circles, and then divide each circle by varying φ at a search step length determined by the candidate θ of the circle. For attitude determination application with a constant baseline length, the candidate position point set only needs to be calculated once. According to the traversal sequence, the search step length Δθ for θ needs to be decided before the decision of the search step length Δφ for φ. As for the selection of a proper search step, to ensure the validity of the selected approximate intersection point, the searching steps must be small enough that the closest candidate position point to the true position can be selected as the best one, and can provide correct ambiguities. However, on the other hand, excessively small search step lengths may increase the burden of calculation. Therefore, to balance the reliability and efficiency, the selection of Δθ and Δφ is of great importance. With these considerations in mind, we will discuss the upper bounds of the search step lengths below.

In detail, for the sake of validity, we hope the largest difference for $\frac{1}{\lambda }\nabla \Delta \rho_{rb}^{ij}$ caused by angle (θ and φ) difference between the true value and its nearest candidate is smaller than 0.25 cycle, which means in extreme situations, the largest difference for SD geometric range $\frac{1}{\lambda }\Delta \rho_{rb}^{i}$ should be smaller than 0.125 cycle. If we convert both the unit LOS vector $e^{i}$ and the baseline vector $r_{rb}$ into angle forms, the SD geometric range $\frac{1}{\lambda }\Delta \rho_{rb}^{i}$ can be presented as a function with baseline length and angle variables only. Firstly, to obtain the upper bound of Δθ, we can take a derivative of $\frac{1}{\lambda }\Delta \rho_{rb}^{i}$ with respect to θ, and rearrange the expression using trigonometric expressions:

(21) $$\partial \left(\frac{1}{\lambda }\Delta \rho_{rb}^{i}\right)=\frac{l}{\lambda }\left[\cos\left(\theta \right)\sin\left(el^{i}\right)-\sin\left(\theta \right)\cos\left(el^{i}\right)\cos\left(\varphi -az^{i}\right)\right]\partial \left(\theta \right)$$

where $el^{i}$ and $az^{i}$ are the elevation and azimuth of satellite i, respectively. Since the biggest difference between the true θ and its nearest candidate value is half the search step length, i.e., Δθ/2, we can get:

(22) $$\frac{\Delta \theta }{2}<\left|\frac{0.125\lambda }{l\left[\cos\left(\theta \right)\sin\left(el^{i}\right)-\sin\left(\theta \right)\cos\left(el^{i}\right)\cos\left(\varphi -az^{i}\right)\right]}\right|$$

For the right side of (22), the following inequality always holds, no matter what values the angles are.

(23) $$\left|\cos\left(\theta \right)\sin\left(el^{i}\right)-\sin\left(\theta \right)\cos\left(el^{i}\right)\cos\left(\varphi -az^{i}\right)\right|\leq 1$$

Thus, we can set the universal upper bound of Δθ, as below:

(24) $$\Delta \theta <\frac{0.25\lambda }{l}$$

For the range of Δφ, similarly, we can take the derivative of $\frac{1}{\lambda }\Delta \rho_{rb}^{i}$ with respect to φ as follows:

(25) $$\partial \left(\frac{1}{\lambda }\Delta \rho_{rb}^{i}\right)=\frac{l}{\lambda }\cos\left(\theta \right)\cos\left(el^{i}\right)\sin\left(\varphi -az^{i}\right)\partial \left(\varphi \right)$$

and get the corresponding range of Δφ as:

(26) $$\frac{\Delta \varphi }{2}<\left|\frac{0.125\lambda }{l\cos\left(\theta \right)\cos\left(el^{i}\right)\sin\left(\varphi -az^{i}\right)}\right|$$

Then, the upper bound of Δφ can be set as:

(27) $$\Delta \varphi <\left|\frac{0.25\lambda }{l\cos\left(\theta \right)}\right|$$

From (24) and (27), we can find that the minimum total number of the candidate position points has an approximate quadratic polynomial relationship with the baseline length; thus, the algorithm is more suitable for the ultra-short baseline situation. Furthermore, for the attitude determination system, if there are some other aiding sensors like INS or tilt sensors which can provide pitch information, the position search space can be further compressed, from a spherical surface to a circle determined by the pitch. In this way, we can not only improve the calculation efficiency of the algorithm, but also increase the success rate of the AR method, especially under the harsh environment with few available satellites and strong multipath interference.

## 5. Algorithm Performance Analysis and Acceptance Test Method

We have introduced the principle of the proposed AR algorithm in angle domain. In this part, the algorithm performance and the corresponding acceptance test method will be discussed.

**Figure 3:**
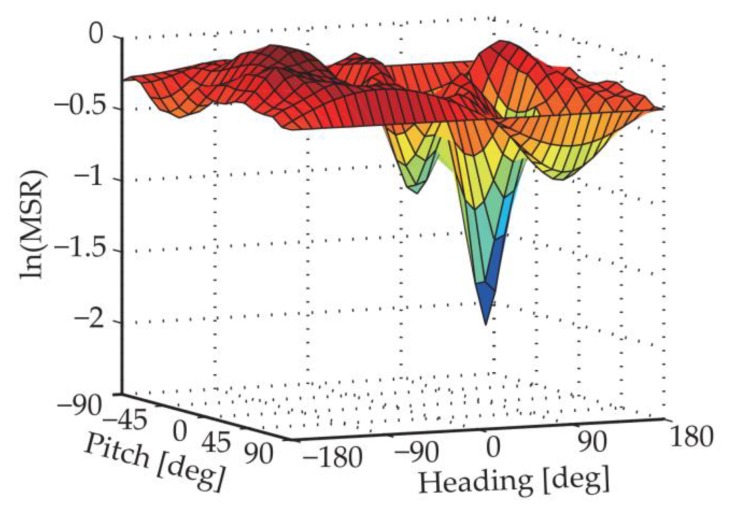
An example of MSR distribution in nature logarithmic form.

The performance of the algorithm, or whether the AR problem can be solved successfully, depends on whether the selection of the approximate intersection point is correct. Figure 3 gives a MSR distribution example in natural logarithmic form. According to the best candidate point selection criterion, we can get the approximate intersection point at the global minimum point. As we can see from the figure, in addition to the global minimum point, there are other valleys, namely local minimums. The MSR distribution can be affected by several factors such as satellite geometry distribution, baseline length and carrier phase noises; in some cases, the global minimum may appear at a wrong point.

**Figure 4:**
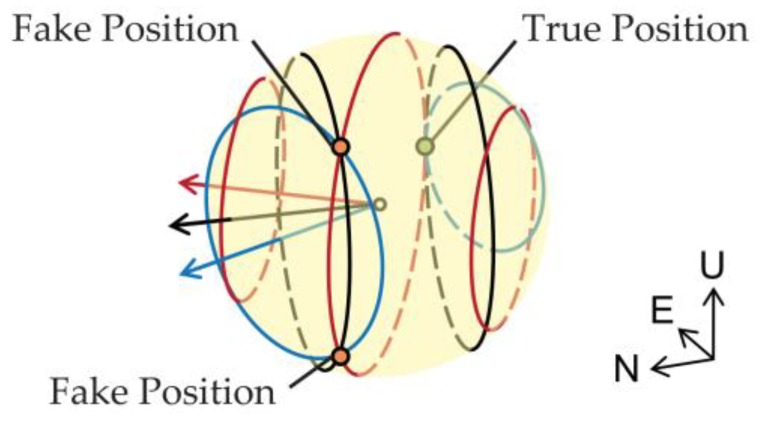
An example for multiple intersection points.

Even for error-free situations, under some special satellite geometry distributions, the phenomenon of multiple intersection points will occur. An example of multiple intersection points is given in Figure 4, where there are three pairs of DD satellites. The baseline length is 0.25 m, and the true pitch and heading are 0° and 90°, respectively. The blue, black and red arrows represent the directions of $\left(e^{1}-e^{2}\right)$,$\left(e^{1}-e^{3}\right)$ and $\left(e^{1}-e^{4}\right)$, respectively, and the elevations and azimuths of the referenced satellite and other satellites are listed in Table 2. The true position point is marked by green, but from the figure we can find that in addition to the true position point, another two intersection points exist. Actually, such a multi-intersection case requires a special integer linear dependence property of the differenced LOS vector set ${\left\{\left(e^{j}-e^{i}\right)\right\}}_{i}$, which happens mostly under the situation where only 4 or 5 satellites are available.

**Figure 5:**
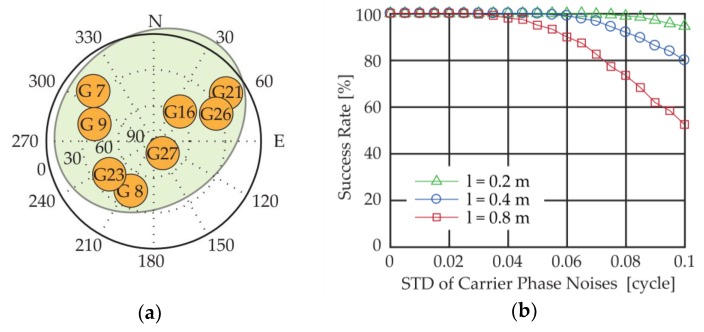
(**a**) Satellite geometry distribution; (**b**) Success rates of the MSR method under (**a**).

To illustrate the effects of different factors on the MSR method, a success rate simulation is conducted, as shown in Figure 5b, with different baseline lengths l, 0.2 m, 0.4 m and 0.8 m respectively, and different carrier phase noise standard deviations (STD) ranging from 0 to 0.1 cycle. For each simulation set, the number of the test samples is 5,000, and the success rate is defined as the percentage of the results that get the correct integer ambiguities. The satellite distribution is plotted in Figure 5a. As we can see from the figure, the success rate drops as the carrier phase noises rise, and the algorithm achieves better performance with short baselines.

**Figure 6:**
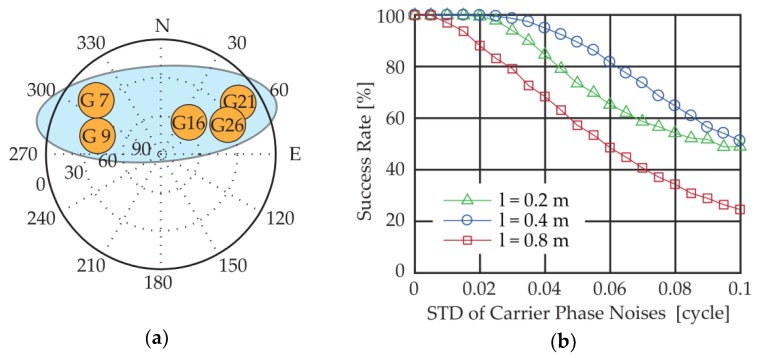
(**a**) Poor Satellite geometry distribution; (**b**) Success rates of the MSR method under (**a**).

Also, we conduct a similar simulation under a poor satellite geometry distribution with only five satellites in view, all of which are in the northern sky. The success rate and the satellite geometry distribution are plotted in Figure 6. With such satellite geometry distribution, the success rate drops greatly in comparison with the situation under normal geometry distribution.

Normally, the shorter the baseline, the higher success rate; but sometimes this is not the case (for example, in Figure 6b, the success rate of l=0.2 m is lower than the one of l=0.4 m), because under extreme poor satellite geometry with few visible satellites, the circles decided by ${\left\{\left(e^{j}-e^{i}\right)\right\}}_{i}$ in two similar directions are less distinguishable for shorter baselines, and thus, increase the difficulty in finding the correct approximate intersection point.

Therefore, with challenges such as poor satellite geometry distribution and large carrier phase noises, the MSR distinction among the local minimums tends to be less obvious, and it is hard to find the correct approximate intersection point. To judge whether the result is valid or not, or strictly speaking, to assess the reliability of the selected approximate intersection point, we propose an acceptance test method based on MSR distinction. It is reasonable to believe that the more obvious the distinction between the smallest valley and others, the higher the probability of finding the correct intersection point. In detail, if $MSR_{1st}$ and $MSR_{2st}$ represent the MSR values of the smallest and second-smallest valleys:

(28) $$\mathrm{Accept}\;r_{rb,1st}\;\mathrm{iff}:\;\frac{MSR_{2nd}}{MSR_{1st}}\geq thres_{MSR}$$

where $r_{rb,1st}$ is the precise baseline vector re-calculated by the integer ambiguities of the smallest valley, and $thres_{MSR}$ is the acceptance test threshold. One important thing to note is that the above MSR ratio is the ratio of the second smallest valley to the smallest valley, not that of the second smallest MSR to the smallest MSR. Sometimes, the points with smallest and second smallest MSRs are neighbors of the true position, which means that the distinction between them may be very small, and both can give the same correct integer ambiguities and be regarded as the correct approximate intersection points. For the selection of the threshold, $thres_{MSR}=1.3$ is recommended, with which the judgment success rate can reach 90% in most ultra-short baseline attitude determination tests we have conducted (an example is given by the multipath experiment in Section 7.1.2).

## 6. Flow Chart of the MSR-Based GNSS/INS Integrated Attitude Determination System

As analyzed in the previous section, the success rate of the MSR method with GNSS is vulnerable to the poor satellite geometry and multipath effects. For attitude determination applications in urban area, challenging environments are inevitable, which makes the availability, precision, and instantaneity of the attitude results hard to guarantee. To settle the problem, our solution is to integrate the MSR-based GNSS attitude determination system with INS.

INS can provide higher precision attitude results at a higher output rate and keep on working for a short while when the GNSS is unavailable. What’s more, INS can output pitch information with which only the traversal in heading is needed for the derivation of the candidate position point set. For the accuracy of the pitch provided by INS, according to the derivation process of Δθ, the absolute error must be less than 0.125λ/l.

**Figure 7:**
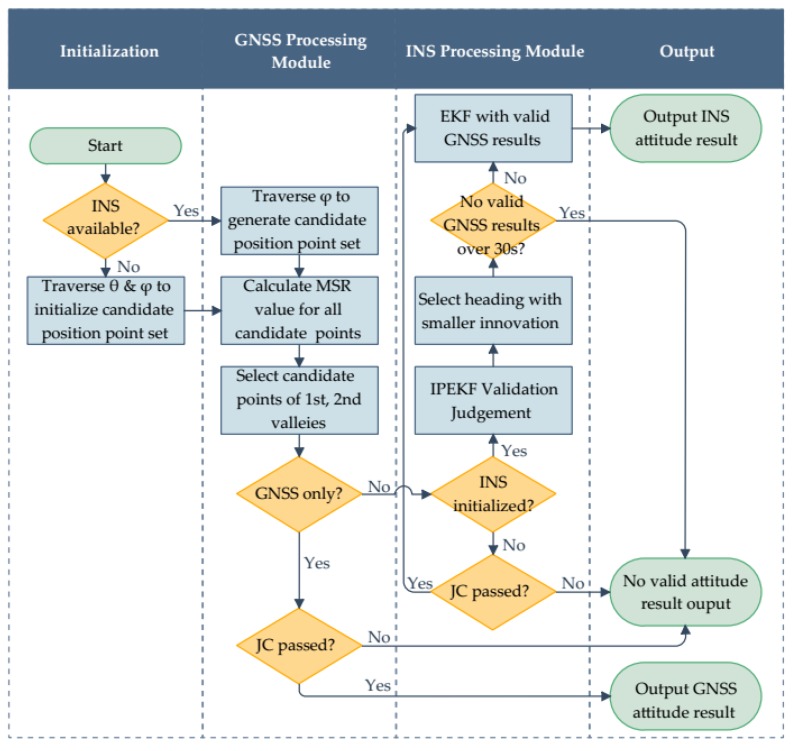
Flow chart of the MSR-based GNSS/INS integrated attitude determination system.

A flow chart of the attitude determination system is depicted in Figure 7. The proposed system contains two main processing modules, GNSS processing module and INS processing module, and supports two attitude determination modes, GNSS only mode and GNSS/INS integration mode.

The main function of the GNSS processing module is to provide various GNSS results, including the positioning, velocity and attitude results. Every epoch, before calculation, satellite selection is made based on satellite elevation mask angle. Then, according to the method described previously, the MSR values are calculated for all the candidate position points which are derived with the baseline length and the pitch if available. Next, we pick up the candidate position points of the smallest and second-smallest MSR valleys, then calculate the MSR ratio between them. To judge whether the GNSS attitude result is valid, the following judging condition (JC), which includes the acceptance test method of (28) and the tests of the prior knowledge, is used:

(29) $$\mathrm{JC}:\;\mathrm{Passiff}:\frac{MSR_{2nd}}{MSR_{1st}}\geq thres_{MSR},\;\left|\left\|r_{rb,1st}\right\|-l\right|<thres_{l},\;\left|\left\|\theta_{rb,1st}\right\|-\theta \right|<thres_{\theta }\;\left(\mathrm{if}\;\mathrm{available}\right)$$

where $\theta_{rb,1st}$ is the pitch calculated by $r_{rb,1st}$, θ is the pitch provided by INS, and, $thres_{l}$ and $thres_{\theta }$ are the thresholds for baseline length and pitch, respectively.

In our system, the traditional loose coupling method [20] is adopted to calibrate INS with the results obtained from the GNSS processing module. To enhance the reliability of the INS output, the validity of the GNSS results needs to be judged. Although the acceptance test method based on the MSR ratio has a low missing detection rate, the false alarm rate is relatively high. Sometimes, with a poor satellite geometry distribution and strong multipath effects, the candidate position point of the second-smallest valley, not the smallest one, is the correct, needed intersection point. Thus, to get more valid GNSS attitude results, we leave two candidate position points, namely, those of the smallest and second-smallest valley, for selection, and calculate their precise headings. Then, we adopt the Innovation Pre-filtering based Extended Kalman Filter (IPEKF) [21] to judge the validity of these two candidate headings, along with the other GNSS results which will be used in the subsequent filtering process.

The state and observation equation are given as follows [20]:

(30) $$x_{k}=F_{k-1}x_{k-1}+\omega_{k-1}$$

(31) $$y_{k}=H_{k}x_{k}+\nu_{k}$$

where $x_{k}={\left[\begin{array}{ccccc} r_{I,k}^{T} & v_{I,k}^{T} & \alpha_{k}^{T} & x_{a,k}^{T} & x_{g,k}^{T} \end{array}\right]}^{T}$ represents the INS state of epoch k, composed of the position vector $r_{I,k}$, velocity vector $v_{I,k}$, attitude vectors $\alpha_{k}$, accelerometer biases $x_{a,k}$ and gyroscope biases $x_{g,k}$, $y_{k}={\left[\begin{array}{ccc} r_{G,k}^{T} & v_{G,k}^{T} & \varphi_{k} \end{array}\right]}^{T}$ represents measurements of epoch k provided by the GNSS processing module, composed of the position vector $r_{G,k}$, velocity vector $v_{G,k}$ and heading $\varphi_{k}$, $\omega_{k-1}$ and $\nu_{k}$ represent system and measurement noises, respectively, $F_{k-1}$ represents the transition matrix whose expression can be found in Section 11.6 of [20], and $H_{k}$ represents the measurement matrix.

The basic idea of IPEKF-based judgment is to test the consistency between the measurements and the state estimators with the use of the following measurement innovation, which is given by:

(32) $$\delta y_{k}^{-}=y_{k}-H_{k}{\hat{x}}_{k}^{-}$$

where ${\hat{x}}_{k}^{-}$ is the EKF predicted state estimator derived from ${\hat{x}}_{k-1}$.

For the two candidate headings, we will select the one with a smaller innovation value for the subsequent validity judgment and EKF process. Before we judge the validity of each measurement innovation component with a certain threshold, the following normalization needs to be done:

(33) $$z_{k,j}^{-}=\frac{\delta y_{k,j}^{-}}{\sqrt{c_{k,j,j}^{-}}}$$

where $\delta y_{k,j}^{-}$ is the jth element of $\delta y_{k}^{-}$ and $c_{k,j,j}^{-}$ is the jth diagonal element of the innovation covariance matrix $C_{k}^{-}$ which is calculated by:

(34) $$C_{k}^{-}=H_{k}P_{k}^{-}H_{k}{}^{T}+R_{k}$$

where $P_{k}^{-}$ and $R_{k}$ is the covariance matrixes of the predicted state estimators and the measurement noises in EKF process, respectively. If the normalized innovations exceed the thresholds, the corresponding measurements will be judged invalid, and the corresponding state components won’t be filtered at this epoch.

Note, for the initialization stage without reliable INS predicted information, JC of (29) is adopted to judge the validity of the GNSS results. Once calibrated, INS can keep providing valid results for a short time, even losing the aid of the GNSS; however, errors will increase as time goes by. Therefore, in our designed system, if the time of losing valid GNSS results exceeds 30 s, INS will not output heading results any longer; after that, once there are new GNSS results (JC passed) available, the initialization process needs to be re-performed.

## 7. Experiment Results

Several static/dynamic experiments in GNSS only or GNSS/INS integration mode under different environments were conducted to evaluate the practical performance of the proposed method. In our system, to realize GNSS attitude determination, two Unicore^®^ UM220-III H GNSS modules were employed, along with two SenseStone^®^ GNSS built-in active antennas with double feed points. For INS part, Xsens^®^ MTi-3-8A7G6 INS module was employed. The raw data can be transmitted in RTCM 3.2 format by the GNSS modules. The message types of the measurements and ephemeris we used are listed in Table 3.

In the experiments, the satellite elevation masks were set as 15°. To better verify the correctness of the results and make an analysis on the measurement performance of the low-cost antennas and receivers under different environments, reference baseline values (for static experiments only) was obtained based on the long-term observation results from the TRIMBLE^®^ NETR9 multi-frequency geodetic-grade receivers in advance. For short baselines, the measurements in DD form are considered to be free of the ephemeris, atmospheric, and clock errors. Hence, if we calculate the DD geometric ranges based on the precise reference baseline and subtract them from the DD measurements, the obtained residuals can be regarded as noises from multipath and receivers only. With the DD residuals, we can evaluate the measurement qualities of the patch antennas and low-cost receivers that we used under different environments.

### 7.1. Experiments with GNSS Only

In this part, the performance of the proposed system with GNSS only was tested.

#### 7.1.1. Experiments with GNSS Only in Open Area

**Figure 8:**
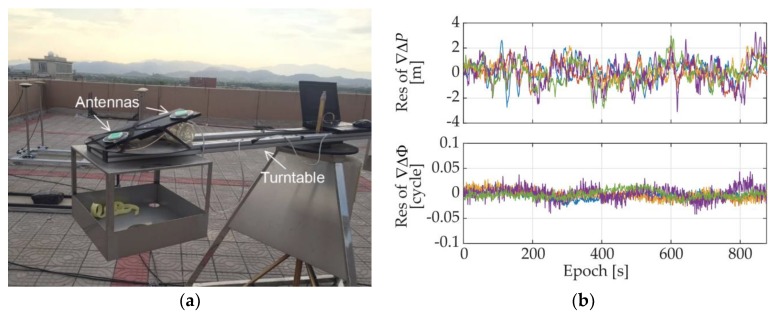
(**a**) Open area environment; (**b**) DD measurements residuals under open area.

Firstly, the static experiment was performed in an open area. The antennas were placed on the arm of a turntable, as shown in Figure 8a, with a baseline length of 0.267 m and a certain pitch. The DD residuals of the measurements were calculated to assess the code pseudorange and carrier phase quality of the low-cost antennas and receivers under the open area, which are given in Figure 8b. The number of the available GPS satellites was 6, and by statistical analysis, the standard deviations of DD code pseudorange noises range from 0.70 m to 1.07 m and the ones of DD carrier phase noises were less than 0.013 cycle.

**Figure 9:**
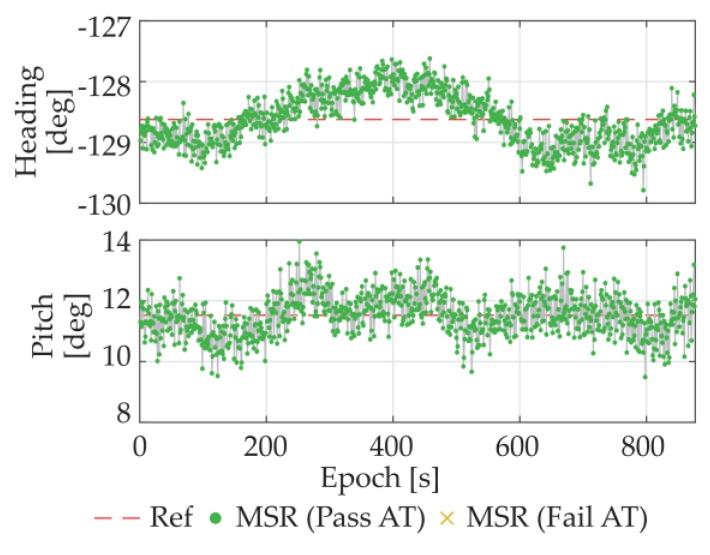
Attitude results of the static experiment under open area.

The heading and pitch results of the proposed MSR based algorithm are plotted in Figure 9, in which the green/yellow points present the results that pass/fail the acceptance test method (AT) of (28). The red dashed lines in Figure 9 are the averages results of TRIMBLE^®^ NETR9 multi-frequency geodetic-grade receivers, which are used as reference value here. As we can see from Figure 8, the pseudorange and carrier phase measurement noises of the low-cost receivers in open area are also quite small. The success rate of the MSR method was 100%, and the standard deviations of the headings and pitches are 0.42°and 0.68°, respectively.

**Figure 10:**
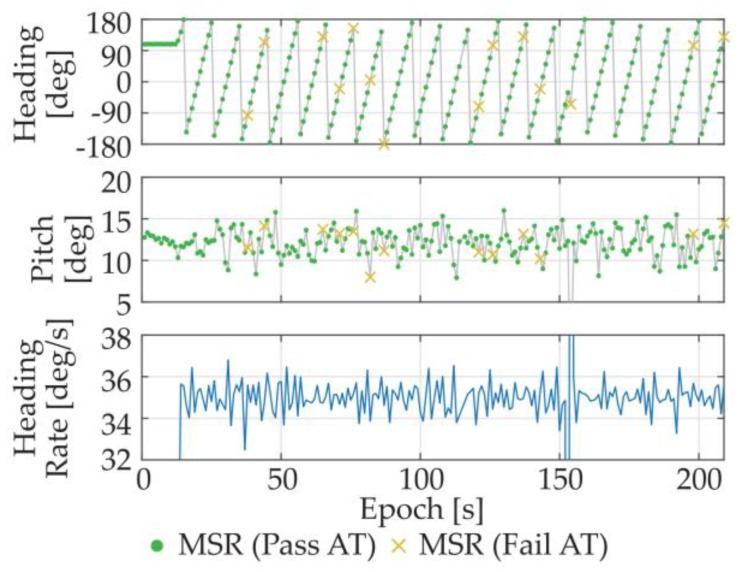
Attitude results of the dynamic experiment under open area.

Then, a dynamic experiment was conducted at the same place. This time, the turntable was moving at a rotational speed of about 36°/s. The attitude results are plotted in Figure 10. According to the angular rate converted from the heading results, the obtained attitude results were consistent with what we set.

#### 7.1.2. Experiment with GNSS Only under Multipath Environment

**Figure 11:**
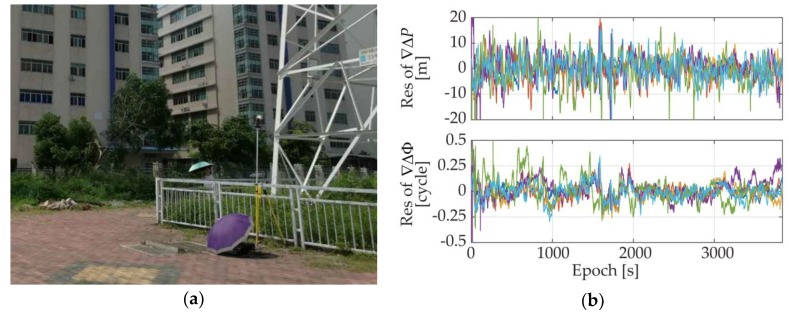
(**a**) Multipath environment; (**b**) DD measurement residuals under a multipath environment.

As one of the applications for which we want to adopt the proposed system, the situation of the attitude determination application for the base station smart antennas was simulated. We conducted a static experiment under multipath environment and placed the GNSS antennas next to a pylon, as shown in Figure 11a. The DD residuals of the measurements under such situations were given in Figure 11b. As we can see from the figure, under such an environment, both code pseudorange and carrier phase measurement qualities deteriorated dramatically, but the carrier phase accuracy was still within the tolerable range. The standard deviations of the DD pseudorange measurements ranged from 3.8 m to 6.7 m, while the ones of the DD carrier phase measurements ranged from 0.07 cycle to 0.12 cycle.

**Figure 12:**
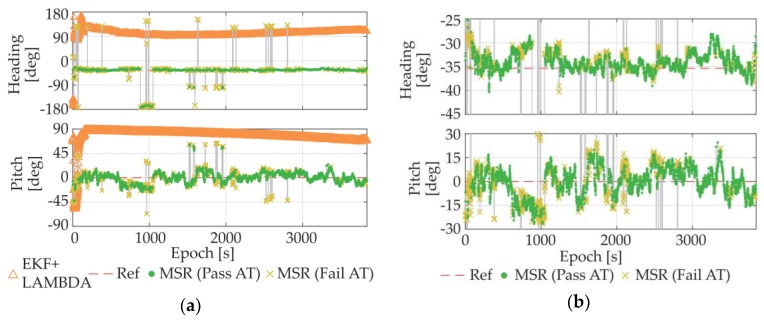
(**a**) Attitude results of LAMBDA+EKF and MSR; (**b**) Enlarged view of MSR results.

In addition to the MSR method, the LAMBDA-based methods were also tested this time. The attitude results of the MSR method and the LAMBDA method that uses EKF to estimate float ambiguities [22] are plotted in Figure 12a. For the LAMBDA method, the traditional ratio-test method (10) was adopted as the acceptance test method with a threshold of 2 [17].

As we can see from the figure, affected by the large pseudorange noises, the float ambiguity estimator could hardly converge, even with the use of EKF, which made the traditional LAMBDA method unable to fix the integer ambiguities. In contrast, our single-epoch MSR method relies on carrier phase only, and thus, could still provide correct results for most of the test time, despite of the attitude precision decline due to the multipath noises and the limit of the ultra-short baseline.

Also, we made a comparison with the results of the single-epoch LAMBDA method, along with single-epoch LAMBDA methods with tight/soft constraints (TC/SC-LAMBDA) [4,19]. The results are listed in Table 4, including the total success rates of all results, the fix rates based on the corresponding test methods, and the success rates of the fix results. As shown by the table, compared with the LAMBDA-based methods, our MSR method could provide a much higher single-epoch success rate under the multipath environment, and the judgment of the corresponding acceptance test method was also more reliable.

### 7.2. Experiment with GNSS/INS Integration

The GNSS-only mode is more suitable to the applications that have low requirement of instantaneity. However, considering the challenges like multipath effects and satellite availability, the requirement of instantaneous output is hard to guarantee with GNSS only for the dynamic applications in urban environments; thus, the GNSS/INS integration mode is more recommended.

**Figure 13:**
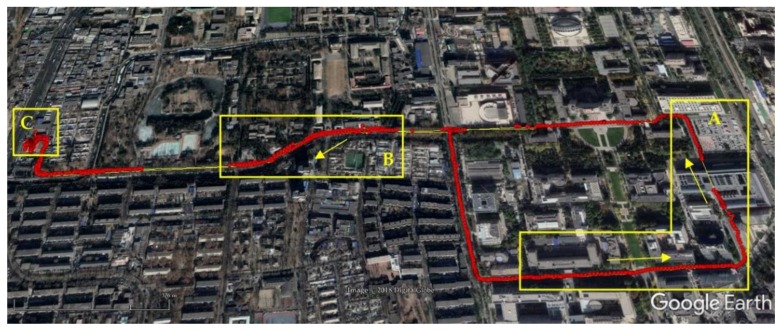
Route of dynamic GNSS/INS integration experiment.

**Figure 14:**
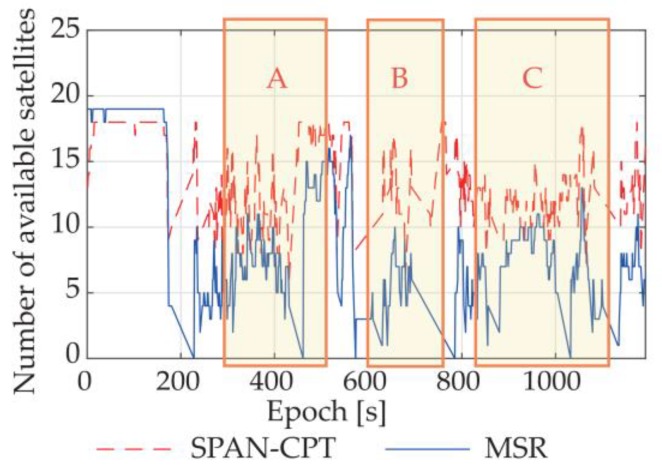
Available satellite numbers of SPAN-CPT and MSR.

**Figure 15:**
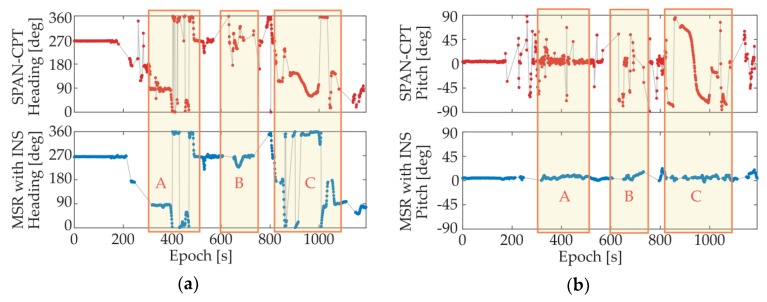
Attitude results of SPAN-CPT and MSR with INS integrated system. (**a**) Heading; (**b**) Pitch.

A dynamic experiment with GNSS/INS integration was conducted in an actual urban environment with surrounding trees and buildings. The moving route is given by Figure 13. To better evaluate the performance of the proposed system, the attitude results of NovAtel^®^ SPAN-CPT high precision GNSS/INS receiver were also collected at the same time. The available satellite numbers and attitude results of SPAN-CPT and the proposed system are plotted in Figure 14 and Figure 15, respectively.

As we can see from the figures, for the dynamic situation in urban environment, the availabilities of both systems declined to a certain extent. Still, the advantages of the proposed system can be observed. For comparison purposes, three route segments have been marked in the figures, and we can find some obvious mistakes in the attitude results of SPAN-CPT, including a two-minute sustained wrong output in Route Segment C. In contrast, our proposed system provided a more stable output with fewer anomalies, even though we realized the system with the use of the low-cost antennas and receivers, and had fewer available satellites for calculation. From Route Segment B, we can find that the INS part was re-initialized quickly and accurately with the MSR-based GNSS attitude determination method, which has a higher success rate for single-epoch solutions.

**Figure 16:**
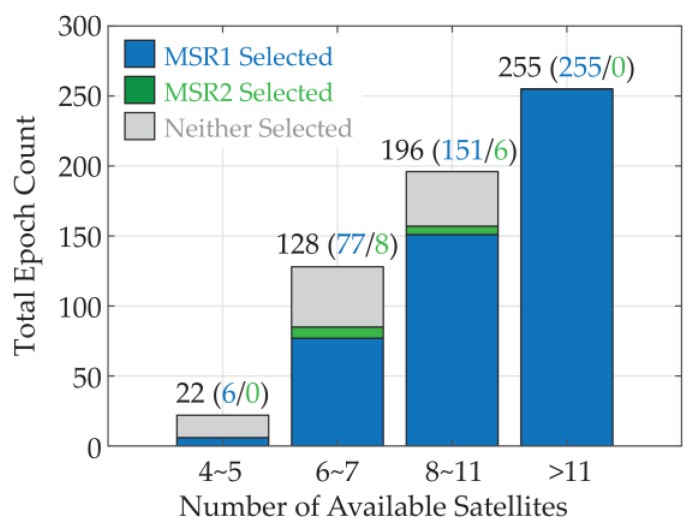
Satellite availability and the IPEKF attitude selection results.

The introduction of the IPEKF further enhances the robustness of the whole system, and reduces the adverse effects of the instability of the GNSS output. Figure 16 gives the IPEKF attitude selection results under different satellite availability conditions during the test. The total number of the epochs that can provide more than three available satellites is 601. For the situation with sufficient satellites, the attitude results with the smallest MSR value still had high reliability, but for the situation with less satellites, sometimes the attitude results of the second-smallest MSR valley were correct. With the aid of the INS and IPEKF, it is much easier to select the correct results or give a validity judgment for the results, and thus, enhance the robustness of the whole system.

**Figure 17:**
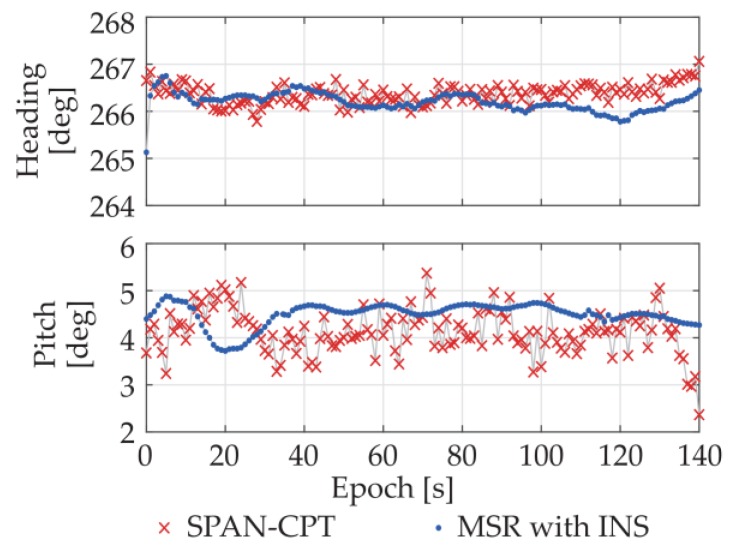
First 140-s static attitude results of MSR-based GNSS/INS integrated system.

Figure 17 shows the first 140-s static attitude results of the MSR-based GNSS/INS integrated system under multipath environment, along with the SPAN-CPT results for comparison purpose. As we can see, benefiting from the INS integration, the output attitude precision of MSR-based system was greatly improved and higher than the one of SPAN-CPT. The heading and pitch standard deviations of the MSR-based GNSS/INS integrated system are 0.20° and 0.25°, respectively.

## 8. Conclusions

To settle the AR problem for ultra-short baseline attitude determination with low-cost GNSS receivers under challenging environment, we proposed a single-epoch AR algorithm, namely MSR, in the angle domain. Unlike traditional algorithms in the ambiguity domain, MSR method neither estimates the float ambiguities nor uses the code pseudoranges, relying rather on carrier phases only which are less affected by multipath; thus, it has a certain adaptation to challenging environments. The performance of the MSR method and the extension MSR-based GNSS/INS integrated attitude determination system for urban dynamic applications under different environments with the low-cost devices has been validated. The experiment results show that the proposed systems could provide a higher success rate and reliability when using low-cost receivers even under the challenging environments.

The above discussion and experiments only involves two degrees of freedom of attitude with one baseline, but the application can be easily extended to three-degree-of-freedom situation with two baselines, which can be used for UAV attitude determination, and will be tried in our future works.

## Figures and Tables

**Table 1:** Satellite geometry distribution for Figure 2.

	Sat 1 (Ref)	Sat 2	Sat 3	Sat 4
**Elevation**	90°	45°	75°	60°
**Azimuth**	0°	0°	180°	240°

**Table 2:** Satellite geometry distribution for Figure 4.

	Sat 1 (Ref)	Sat 2	Sat 3	Sat 4
**Elevation**	60°	60°	60°	60°
**Azimuth**	0°	60°	120°	180°

**Table 3:** Results of different algorithms under multipath environment.

System	Measurement Message Type	Ephemeris Message Type
GPS	1074	1019
BDS	1124	63

**Table 4:** Results of different algorithms under multipath environment.

Algorithm	Success Rate (All)	Fix Rate	Success Rate (Fix)
LAMBDA	1.04%	12.61%	0.41%
TC-LAMBDA	6.36%	18.94%	9.35%
SC-LAMBDA	3.67%	5.94%	0.44%
MSR	91.48%	83.38%	95.91%
